# Notes and News

**Published:** 1887-01-15

**Authors:** 


### NOTES AND NEWS.

COLLECTED AND COMMUNICATED.

THE Duke of Westminster and other friends of the Hospital for Women, Soho Square, have recently presented a petition to Her Majesty in Council praying for a Charter of Incorporation. The petition will be taken into consideration by a Committee of the Council on the 12th prox.

THE late Mr. George Fielder, of Leatherhead, has left £500 Consols to each of the following hospitals: Charing Cross, Middlesex, Surrey County, Seamen's, and the London Homœopathic.

MR. A. E. SHIPLEY, Demonstrator of Comparative Anatomy at the University of Cambridge, and Scholar of Christ's College, has just been elected to a junior fellowship.

THE thirteenth annual meeting of the Hospital Saturday Fund is to be held at the Memorial Hall on the 8th prox. Lord Brabazon has promised to preside.

DR. ERNEST S. REYNOLDS has been appointed, for two years, resident medical officer of the Royal Infirmary, Manchester. In memory of the late Mr. Alderman T. Rose, who left the Infirmary £10,000, it has been decided to name one of the wards the "Thomas Rose" Ward.

THE Bristol Eye Hospital is in search of a sum of £600 to clear the expenses in connection with the new premises.

A MOVEMENT is on foot in Glasgow to erect a hospital, to be called the Queen's Infirmary, in the Queen's Park, commemorative of the Jubilee.

THE chairman of the York County Hospital intends to move, at the next quarterly meeting of the governors, that the York Eye Institution be amalgamated with the York County Hospital.

THE Hastings Hospital Sunday Fund collection will be made, as in previous years, on the last Sunday in January.

MR. E. G. PAPWORTH has executed a bust of the late Dr. Russell for the Birmingham General Hospital, subscribed for by the medical profession of that town.

THE whole of the profits resulting from the sale oi Mr. Lewis Carroll's new book, *Alice's Adventures Underground*, are, we learn, to be devoted to hospitals and convalescent homes for sick children.

MISS ROBERTSON, late Matron of the Sussex County Hospital, Brighton, has accepted the appointment of Lady Superintendent of the St. Helena Home, 1, Grove End Road, N.W.

No less than 250—rather over a third—of the beds at Guy's are now closed, and must remain so until the hospital is relieved from its present financial difficulties.

MR. R. G. SALMOND'S well-known appeal on behalf of the British Home for Incurables—"One Million Shillings required by the end of the year"—has been met, during the past twelvemonths, by a contribution of 513,857 shillings; a trifle over half the number asked for!

THE concert recently given at the Kilmarnock Corn Exchange, Hull, in aid of the Fever Hospital and Infirmary, resulted, after payment of working expenses, in a sum of £65 being handed to the treasurer.

THE new medical college, built in connection with the London Hospital, is to be opened next Easter by the Prince and Princess of Wales.

A DANCE took place on 6th inst., at the Portman Rooms, in aid of the Great Northern Central Hospital, and proved a great success. The orchestra was conducted by Miss May Ostler.

A SUGGESTION has been gaining ground in Woolwich, that a cottage hospital would be an appropriate memorial of the Queen's Jubilee in that town.

THE Fancy Fête held at Torquay at Christmas in aid of the Western Hospital for Consumption, proved highly successful, and was patronised by many of the best-known residents in the neighbourhood. The Fête realised something like £200.

MRS. HAINS, of Alton House, Plymouth, has given the munificent donation of £5,000 to the South Devon and East Cornwall Hospital, for the purpose of adding a memorial wing to that institution.

THE employés of Messrs. Barclay, Perkins and Co., have sent the secretary of the Hospital Saturday Fund a cheque for £20, towards the Morley House Seaside Convalescent Home, an excellent little institution whose existence is due to one of the best suggestions Mr. Frewer ever made.

MR. CHARLES JORDAN has presented to the Manchester Royal Infirmary memorial busts of Charles White and Joseph Jordan, two distinguished surgeons of that town. The pedestals are inscribed—"Charles White, F.R.S., one of the Founders of, and for thirty-seven years a Surgeon to, this Charity. Born 1728; died 1813"; and "Joseph Jordan, F.R.C.S., Founder of the first recognised School of Anatomy in Manchester, and surgeon to this Charity. Born 1787; died 1872."

SEVERAL improvements have just been caried out at the Bristol Royal Infirmary. The new Nurses' Home has proved a great boon, and the health of the nurses has in consequence greatly benefited.

THE recent extension of the Bootle Borough Hospital has furnished the town with an institution worthy of its present importance. The leading residents have contributed liberally to the good work, and the cost of the work has, we believe, now been covered. A special effort is being made to furnish the new building.

---

THE following vacancies are announced this week, the amount of the salary being placed in each case after the name of the institution :—

**Honorary Medical Officer.**

St. Pancras Dispensary. Physician.

**Resident Medical Officers.**

Worcester Infirmary. House Surgeon, doubly qualified.—£100, with board.
Flintshire Dispensary, Holywell. One, registered.—£100, and furnished house.
Chorley Dispensary. House Surgeon and Apothecary, doubly qualified —£130, with house.
Bedford Infirmary. One, doubly qualified.—£100, with board, etc.
Scarborough Hospital. House Surgeon and Acting Secretary, registered.—£80, with board, etc.

**Non-Resident Medical Officers.**

Chelsea Union. One.—£100.
Lampeter Union. One, registered.—£30.

**Matrons.**

Ledbury Cottage Hospital.—£25.
Southampton Nurses' Institute. Medical training preferred.—£40, with board, etc.

**Trained Nurses.**

Canterbury Nursing Institute. One.
Bradford Infirmary. One.—£25, with uniform.
Huddersfield Infirmary. One, night.—£22, with uniform.

**Probationers.**

Canterbury Nursing Institute. Several.

---

THE Mayor and Corporation of Folkestone have had under consideration the question of the celebration of the Queen's Jubilee. The most appropriate form for it to take will, they think, be in the improvement of the hospital accommodation of the town.

---

THE local medical officer of health strongly advises that the Aberdeen City Hospital should be enlarged. This institution, it appears, affords at the present time accommodation for only sixty-four beds; but the medical officer is of opinion that there should be about one bed for every thousand inhabitants, equivalent to an aggregate of about 120 beds.

---

"A GOOD name," says an Eastern poet, "is as priceless as the pearls of the sea." Mrs. Rahn, who has just retired after many years service as matron of the East Suffolk Hospital, has carried away with her the cordial esteem of everyone connected with that institution. Even the Christmas decorations were made to pay testimony to her worth. In the surgical ward, for example, were suspended the words, "Our late Matron, may good wishes and blessings attend her"; and even in the kitchen was a banner, inscribed, "May the loss we all feel for our late Matron bring blessings on her head."

---

THE recent Hospital Sunday Fund collection in Norwich was eminently successful, the contributions amounting to £1,061, the largest sum which any one year has yet produced in the county town of Norfolk. The amount showed an increase of £78 over the preceding year. Norwich is justly proud that it has got into "four figures."

---

THE town of Chard is incubating a scheme for the loyal celebration of the approaching Jubilee. What the result of the process may be is as yet a little doubtful. The number and variety of the suggestions made indicate considerable versatility of taste and judgment among the Chardians. A cottage hospital heads the list: and is followed by—a permanently engaged professional nurse; third in order is a statue—of whom, it is not stated. Are the good people ambitious of a stone effigy of Her Majesty herself? A free library is also suggested; then a public playground. Still more ideas flow in—for example: public swimming-baths; an extension of the town's water-supply; and finally, a brand-new, or, failing that, a *second-hand organ* for the Corn Exchange. Whether the organ, in addition to being *second-hand*, is to have a proper keyboard, or to be of the *barrel* variety, with a turning handle, is not stated. If we might make bold to advise, we would suggest that either a cottage hospital or a public playground should have the preference.

---

THE "elephant man" is to remain as a permanent inhabitant of the London Hospital. This is in every way satisfactory. The miserable curiosity of a prurient public will thus be deprived of one spectacle at any rate. Let us be thankful for so much.

---

THE Edinburgh Royal Infirmary has just issued its annual report. It is gratifying to observe, among other things, that whilst the number of in-patients treated during the year has considerably increased, there has been a decided decrease in the expenditure. It is also worthy of note that two wards have been given to Dr. Keith, and that ovariotomy cases, in Edinburgh at any rate, are not to have a *special* hospital built for them. For twenty-five vacancies occurring in the nursing department there were over 500 applicants. The new wards, which will be opened immediately, will raise the number of beds in the hospital to 660. The Infirmary has a library of 4,300 volumes. It is of interest to state that the average cost per bed during the year was—for food, £20 13*s*. 7¼*d*.; for medical necessaries, £6 10*s*. 8½*d*.; for wine, 3*s*. 3¾*d*.; and for spirits, 8*s*. 10¾*d*.

---

A DAILY contemporary states that the annual income of the London hospitals may be set down roughly at £500,000, whilst that of the various foreign missionary societies is nearly twice that sum. There are people, who never give to either of these causes, who will pretend to be very much disgusted at the contrast. Our recommendation to Londoners would be to diminish their extravagant expenditure on things superfluous and injurious, and so double the incomes both of the hospitals and the missionary societies.

---

A PROPOSAL has been made, as a memorial of the Queen's Jubilee, to extend the North London Hospital for Consumption. This valuable institution, which is situated in the bracing neighbourhood of Mount Vernon, Hampstead, was originally designed to provide for 110 patients, but, financial difficulties arising, the coat had to be cut according to the cloth. The present seems an excellent opportunity for carrying out the original intention. Patients now seek this institution from all parts of the country, and there is, we believe, a growing opinion that this disease, both in its incipient and in its more advanced stages, can be better battled with in a place where the air is dry and bracing than it can where the climate is mild and less invigorating.

---

"A TOWN without a hospital is like the sky without the sun." These words of Cardinal Manning's we had occasion to quote in our first number, and again there comes an instance of their application. At Hayward's Heath, the child of a working-man recently fell on a fire, and received terrible injuries. As Hayward's Heath does not possess a cottage hospital, it was decided to take the little sufferer to the Children's Hospital at Brighton, but the friends in charge of it, on arriving at the station, found, to their dismay, that they had *two hours to wait* for the next train due to stop at Hayward's Heath! Surely there is room for a little cottage hospital at such a place as this.

---

So unexpectedly great was the success of the recent Hospital Bazaar at Hastings, that the ladies who organised it, intend to arrange another to effect the final clearance of the debt. The incubus of despondency which weighed so heavily on certain members of the Building Committee a little while ago, has vanished as if by the touch of some fairy's wand.

---

A FRUGAL Scotchman going to London for the first time was heard to exclaim to a friend on his return, that he had not been in the great city "twa hoors" before "bang went saxpence." Without pausing to discuss the truth of the story, we may observe that there is no doubt that the Scotch *do* possess the knack of making a little go a long way. The annual report of the Edinburgh Provident Dispensary displays a really surprising list of achievements. The total income of the Dispensary during the past year was only £187 10*s*., but with these small means, this little Association, whose existence is perhaps unknown beyond its own borders, managed to prescribe for no less than 3,902 patients, involving 12,000 consultations; while 2,555 patients were seen at their own homes, the latter involving 12,000 visits. Then there were 355 infectious cases, 783 teeth extractions and minor operations in surgery, 71 cases of diseases of women, and 399 vaccinations. "The result for the outlay," remarked the chairman at the annual meeting held a few days ago, "seems to me stupendous." So it undoubtedly does to us.

---

THE Lord Mayor, the President of Guy's, and *The Times*, all went wrong touching the civic position of the founder of the great Borough hospital. Thomas Guy never was an "Alderman of the City of London," nor is it likely that a man of such penurious and frugal habits would have cared for the aldermanic gown. It was the custom of the old Lombard Street bookseller, says Nichols, for him to dine at his counter every day "with no other table-cloth than an old newspaper." We can scarcely conceive an alderman so simple in matters gastronomic as to use his *Intelligence* or his *Newes* as a substitute for a linen coverlet.

---

THERE are many directions—some almost unthought of—in which a little practical assistance may be rendered to the hospitals. The Rev. W. H. Bruce, the head master of the Earl's Court School, has, for some time past, allowed his boys to organise little dramatic performances in aid of the Hospitals for Sick Children, resulting in about £60 being raised—enough to keep one little cot for two years, be it remembered. On the 5th and 6th instant, the boys gave another of their really clever dramatic entertainments at the St. Matthias' School-rooms, Warwick Road, Kensington; which on both nights brought together crowded attendances, including a very large sprinkling of the *élite* of the little hamlet where for so many years John Hunter lived and experimented. The proceeds of these two entertainments are to go in their entirety to the Royal Victoria Hospital for Children at Chelsea.

---

THE new hospital which Mr. J. G. Hubbard, M.P., is about to give to Nottingham, is now very nearly ready for opening. The institution, which is called the Nursery Hospital, has cost about £4,000, Mr. Hubbard has built the hospital as a thank-offering in remembrance of the happy escape from sudden death of Mr. Egerton Hubbard, when riding into Buckingham after his victory over Sir Harry Verney in 1874.

---

THE Ilkley Bath Charity Hospital has received a legacy of £1,000 under the will of the late Mr. Thomas Emsley of Burley. The money has been wandering in search of its lawful exchequer. The deceased gentleman left it to "The Ilkley Convalescent Hospital," and the executors, thinking that the "Semon Convalescents' Home" at Ilkley was intended, first paid the legacy over to the Bradford Corporation, who are the trustees of that institution.

---

THE good people of Ashbourne propose to celebrate the Queen's Jubilee by the erection of a Cottage Hospital, called the "Victoria," which they hope will emulate the success of the institution at Leek, though on a smaller scale. Ashborne is certainly not very well off for hospital accommodation, for at present cases have to go either to the Cottage Hospitals at Leek and Wirksworth, or to the Infirmary at Derby.

---

"CHRISTMAS" has been celebrated somewhat late at some of our hospitals, but the festivities have been none the less enjoyable. On the 6th inst., Miss Winn, the Matron of the Hospital for Diseases of the Throat, Golden Square, gave her customary annual entertainment to the patients and servants. A monster Christmas-tree was soon denuded of its pretty presents, one of which was destined for the matron herself, and had been subscribed for by the patients and staff. The evening was brought to an agreeable close with a conjuring entertainment, and a little music and song.

On Thursday evening, the 6th inst., the usual fortnightly entertainment was given to the in-patients of the Cancer Hospital at Brompton. About sixty patients and nurses were present, and also many visitors. Among them we noticed the Rev. N. Liberty, Chaplain to the Hospital; Dr. Marsden, the Senior Surgeon; Mr. Jessett, Mr. Hughes, the Secretary, and Mr. Clark. Two pieces were performed, *May Blossoms* being the first, in which the principal parts were well given by Mr. H. Stopford Ram and the Misses Haffenden. In the after-piece, a musical version of the farce of *Chiselling*, Mr. M. H. Cotton acted admirably, and was very comic indeed. Mr. Herbert Walther also contributed in no small degree to the success of the entertainment. Mr. Ram, who sang some capital comic songs, kindly superintended the whole of the performance. These evenings are greatly enjoyed by the inmates of this excellent charity.

---

Last Saturday, the third of a series of entertainments to the in-patients of the Markham Square, Chelsea, branch of the St. John's Hospital for Skin Diseases, took place under the direction of Dr. C. M. Campbell. The patients and visitors appeared heartily to enjoy the capital programme which had been provided by the doctor. Mr. Hirwin Jones—quite an apostle of vocal melody—gave several really splendid songs, especially Temple's "'Tis all that I can say." Miss Rossi's recitations displayed great ability in an art which few ladies attempt. Mlle. Marianne Eissler's violin solos delighted the patients, as also did Signor Carlo Ducci's marvellous pianoforte performances. Madame Mathilde Zimeri sang very sweetly. Between the parts she went among the patients, accompanied by Dr. Campbell, and made several kind inquiries as to their health. The little ones appeared specially to attract Madame's attention, and her kind and sympathetic words to them won the genuine appreciation of the sufferers. A cordial vote of thanks was passed to Dr. Campbell, who, in acknowledging it, said it was due rather to the artistes who had helped him than to himself.

---

Some few weeks since we referred to the critical condition of the finances of the Newcastle Infirmary. The Committee which was appointed to inquire into the whole question have made a very careful examination of the accounts, and gathered information from other institutions. From their investigations thus far, it seems that, as regards retrenchment, there is not much room for hope. The deficit now amounts to over £5,000 a year, and this sum will be but little reduced by the trifling "clippings" in the expenditure for which there may possibly be room. Now that it is tolerably clear that there is no flagrant mismanagement or waste, it behoves the Committee to turn their attention to the income of the Infirmary. For many years past the subscription list has remained almost stationary, and this, too, despite the fact that Newcastle has steadily grown in opulence and prosperity. The governors, and, indeed, all who have the welfare of the Newcastle Infirmary at heart, ought to evince a practical sympathy by canvassing among their friends, not only in the town, but along Tyneside generally. The spirit of charity is not dead among Englishmen, if hospital managers would but arouse themselves to a sense of their moral responsibilities by exerting a personal influence among their friends. If this is done at Newcastle, we do not doubt that many hundred new subscriptions may be got in, and the present downward course arrested once and for all.

---

The Hospital Sunday movement in Birmingham seems to require the infusion into it of a little enthusiasm. "Surely" as Mr. John Jaffray, J.P., one of ablest workers in the cause of the Hospitals, well said, "the men of Birmingham are not going to allow their annual Sunday collection to continue to decline in the way it has been doing! The claims of the General Hospital should certainly be sufficient to ensure the upward movement of the fund. The amounts collected during the past three years have been £5,145, £4,773, £4,612. It is not therefore surprising that a suggestion has been made, that the committee to be appointed next year shall seriously take into consideration the cause of this downward course.

---

Two of the principal wards of the Suffolk General Hospital at Bury St. Edmunds have been greatly enlivened and beautified by the substituton of enamelled tiles for the stucco facing which formerly covered the walls. These tiles are of cream colour, relieved by a dado of deep red, while around the top of the room runs a neat and effective border of brown flowers upon a cream-coloured ground. These improvements are due to the generosity of Dr. Image, F.R.C.S., of Herringswell, who from 1843 to 1873 was surgeon to the hospital, and also to that of Mr. Edward Greene, M.P., who has for many years past been a consistent and liberal supporter of the institution.

---

The comfort of the patients is being still further increased by the purchase of spring mattresses for the beds, in lieu of the old straw palliasses, to as great an extent as the somewhat limited funds at the disposal of the committee will permit. There is, however, yet one more radical improvement needed, and that is the provision of a separate ward to be devoted exclusively to the use of children, who are at present accommodated in the adult wards. Has Bury St. Edmunds no Mrs. Gulson, who will take energetic and successful measures to supply this want?

---

The quarterly report submitted at the meeting of the committee of the Belfast Royal Hospital, held last week, disclosed a state of things by no means creditable to a large, thriving, and wealthy town like the "Capital of Ulster." The Royal Hospital is, we believe, in the matter of administration, *sans reproche*. The people of Belfast rush to it in *their* hour of need, but the hospital, in *its* hour of need, is allowed to go begging in the best way it may. The working-classes who use the hospital are the classes who should support it most, but these, to a very large extent, we regret to say, shirk their responsibilities. Many a working-man seems to be possessed with the idea that the hospital should be provided free for him. He cares little, and thinks less, how the money comes to the hospital—whether from the pockets of the rich or from the clouds, so long as there is an institution to which he can go.

---

With this feeling so rampant as it is among the working population, it is small wonder that so many of our public charities suffer from chronic pecuniary embarrassment. At many of our hospitals affairs have now assumed a very serious aspect, and, unless the working-classes of this country can be brought to recognise the fact that "to give is a duty," the crisis which many have long seen to be impending will not be averted. The Belfast institution is now labouring under a debt of nearly £1,550, and in all parts of the country it is now the rule, instead of the exception, to find our hospitals similarly crippled, and the sphere of their noble work "cabin'd, cribb'd, confin'd."

---

Some of the wards of Westminster Hospital have undergone a very pleasing metamorphosis at the hands of the Kyrle Society. Until recent years, hospital walls have borne a striking resemblance to those of the workhouse type—dull, dreary, and monotonous in the extreme; calculated rather to depress than to cheer the poor sufferer. The pretty floral paintings—especially those on the walls of the men's wards—indicate a step in the right direction; and it is to be hoped that the beautiful decorations which have been carried out at Westminster Hospital will speedily be copied by other institutions.

---

The opening of the Cama Hospital for Women at Bombay is another step in the movement popular already in India for providing the native women with doctors of their own sex. The Hindoo woman, it is declared, no matter the character or extent of her suffering, would rather face the extremest torture or even death than break through the traditions of her country by being tended by a man. The prejudice is inborn, and not to be overcome, and therefore Lady Dufferin is devoting her energies in furtherance of the movement for providing Hindoo ladies with doctors of their own sex. In India, ladies who desire to devote their lives to the practice of medicine and surgery will find a field of labour almost inexhaustible in extent. Dr. Edith Pechey and Dr. Charlotte Ellaby have the management of the Cama Hospital.

---

The relations of sisters and nurses to students have always afforded matter for discussion. The reason for this is not far to seek. It is not yet sufficiently realised that there are hospitals and hospitals. At a county infirmary, like Leicester, for instance, the nurses have to attend to most of the dressing, the temperatures, and other similar matters. At a Clinical Hospital, on the other hand, where there are more students than patients, as sometimes happens, it must be seen at once that even the smallest details will have to be entrusted to the students, or much practical work will be lost to them, to their serious detriment. A recognition of these different circumstances would prevent disagreement and loss of temper, besides proving that good nursing often consists as much in tact and the power to recognise, with Milton, that "they also serve who only stand and wait," as in anything that can be mentioned.

---

The *Pall Mall Gazette* lately told the following amusing story of a visit which the "Prince of Jockeys" paid to Sir James Paget:—

"Archer, having been bitten or otherwise injured by a horse on one occasion, called on Sir James Paget. The eminent surgeon having bound up his wound, Archer requested to know how long it would take to heal. 'Oh,' said Sir James, 'I think in three or four weeks you will be all right.' 'But shall I be fit for the Derby?' asked Archer. 'Ye-es,' was the reply. 'Oh, yes! I think you may go to the Derby.' 'No, but you don't quite understand me, Sir James,' persisted the jockey. 'I mean, shall I be fit to ride?' 'Well, I don't know,' was the answer. 'Better drive; better drive!' Archer, rather taken aback by this very innocent and unexpected rejoinder, had to explain. 'I am afraid, Sir James, you scarcely realise who I am?' 'No,' said the surgeon, politely, referring to the patient's visiting-card. 'I see I have the honour of receiving Mr. Archer, but—' 'Well,' said Archer, 'I suppose I may say that what you are in your profession, Sir James, that I am in mine,' and proceeded to tell him what that profession was. The famous surgeon, on learning the status of his visitor, was at once greatly interested, and asked him eagerly many questions, among others—what would be his loss supposing he should be unable to fulfil the Derby engagement? to which Archer replied: 'About £2,000.' His average annual income he stated (if I mistake not) to be about £8,000; upon which Sir James is said to have remarked: 'You may well say that what I am in my profession that you are in yours. I only wish that my profession were half as profitable as yours.'"

---

The President and Managers of the Aberdeen Infirmary, have announced their intention to apply to Parliament next session for an Act to repeal the charters of 1773 and 1852, and to provide for a new incorporation of the present managing body, and for the special appointment of Boards of Directors to control all matters relative to the Infirmary, Convalescent Hospital, and Lunatic Asylum. Powers will also be sought to rebuild, remodel, or alter the existing buildings, or to erect and manage new ones.

---

Dr. Joseph Schneer, of Alassio, will shortly publish a pamphlet concerning the proposal to establish a hospital for consumption in the Riviera di Ponente, the climate of which is well known for its health-giving effects in cases of pulmonary disease. The idea of an International Home or hospital for such cases was originally set forth by Dr. Schneer in a letter to *The Times* in 1884, and the proposed pamphlet will deal with the following questions, viz.: (*a*) the necessity for such an institution; (*b*) the most desirable spot for its erection; (*c*) the method of its organisation; and (*d*) the sum which ought to be raised before any positive beginning can be made. Any suggestions on these points will be welcomed by the author of this undoubtedly beneficent scheme.

